# The Consequences of a BRCA Mutation in Women

**DOI:** 10.6004/jadpro.2015.6.3.2

**Published:** 2015-05-01

**Authors:** Janice Famorca-Tran, Gayle Roux

**Affiliations:** Texas Oncology, Houston, Texas, and Texas Woman’s University College of Nursing, Denton, Texas

**The Consequences of a BRCA Mutation in Women**

A continuing education article for nurse practitioners, physician assistants, clinical nurse specialists, advanced degree nurses, oncology and hematology nurses, pharmacists, and physicians.

**Release date:** May 15, 2015

**Expiration date:** May 15, 2016

**Expected time to complete this activity as designed:** 0.75 hours

**Meniscus Educational Institute**

3131 Princeton Pike, Building 1, Suite 205A

Lawrenceville, NJ 08648

Voice: 609-246-5000

Fax: 609-449-7969

E-mail: lrubin@meniscusedu.com

**Journal of the Advanced Practitioner in Oncology**

37 Main Street

Cold Spring Harbor, NY 11724

Voice: 631-692-0800

Fax: 631-692-0805

E-mail: claudine@harborsidepress.com

© *2015, Meniscus Educational Institute. All rights reserved.*

## Faculty

**Janice Famorca-Tran, RN, MS, AOCNP®, CBCN®, NP-C,** Texas Oncology

**Gayle Roux, PhD, NP-C, FAAN,** Texas Woman’s University College of Nursing

## Activity Rationale and Purpose

Deleterious mutations in*BRCA1* and *BRCA2* genes are the only known causes of hereditary breast and ovarian cancer (HBOC) syndrome. These mutations account for 60% of hereditary breast cancer. Women with HBOC face many decision-making challenges and rely heavily on information and guidance provided by their oncology care providers. Therefore it is imperative that advanced practitioners (APs) in oncology be informed about the many facets surrounding HBOC. The purpose of this article to provide APs with a clinical overview and the most up-to-date information regarding risk assessment for these genetic mutations, genetic testing, psychological consequences associated with genetic testing, the importance of pre- and posttest counseling, and posttest risk-reduction strategies.

**Intended Audience**

The activity’s target audience will consist of nurse practitioners, physician assistants, clinical nurse specialists, advanced degree nurses, oncology and hematology nurses, pharmacists, and physicians.

## Learning Objectives

After completing this educational activity, participants should be able to:

1. Define hereditary breast and ovarian cancer syndrome (HBOC) and the genetic mutations associated with this condition
2. Describe risk-reduction measures for individuals with HBOC and their families that may decrease the risk of developing cancers associated with HBOC
3. List National Comprehensive Cancer Network testing criteria for HBOC
4. Discuss the role of the advanced practitioner in regards to genetic counseling in HBOC
5. Identify existing laws that govern the rights of BRCA-positive individuals
6. Describe some of the psychological issues facing HBOC patients and families

## Continuing Education

**Statement of Credit—Participants who successfully complete this activity (including the submission of the post-test and evaluation form) will receive a statement of credit.**

**Physicians.** The Meniscus Educational Institute is accredited by the Accreditation Council for Continuing Medical Education (ACCME) to provide continuing medical education for physicians.

The Meniscus Educational Institute designates this journal article (2015-005-02-MJ) for a maximum of 0.75 AMA PRA Category 1 Credits™. Physicians should claim only the credit commensurate with the extent of their participation in the activity.

**Nurses.** This activity (2015-005-02-NJ) for 0.75 contact hours is provided by the Meniscus Educational Institute.

The Meniscus Educational Institute is accredited as a provider of continuing nursing education by the American Nurses Credentialing Center’s Commission on Accreditation.

Provider approved by the California Board of Registered Nursing, Provider No. 13164, for 0.50 contact hours.

**Pharmacists.** The knowledge-based accredited education lectures are intended for pharmacists involved in the care of cancer patients. This educational activity is sponsored by the Meniscus Educational Institute.

The Meniscus Educational Institute is accredited by the Accreditation Council for Pharmacy Education (ACPE) as a provider of continuing pharmacy education. The ACPE Universal Activity Number assigned to this program, for 0.75 contact hours, is 0429-0000-15-005-H04-P.

## Financial Disclosures

All individuals in positions to control the content of this program (eg, planners, faculty, content reviewers) are expected to disclose all financial relationships with commercial interests that may have a direct bearing on the subject matter of this continuing education activity. Meniscus Educational Institute has identified and resolved all conflicts of interest in accordance with the MEI policies and procedures. Participants have the responsibility to assess the impact (if any) of the disclosed information on the educational value of the activity.

**Faculty**

**Janice Famorca-Tran, RN, MS, AOCNP®, CBCN®, NP-C,** has nothing to disclose.

**Gayle Roux, PhD, NP-C, FAAN,** has nothing to disclose.

**Lead Nurse Planner**

**Wendy J. Smith, ACNP, AOCN®,** has nothing to disclose.

**Planners**

**Jeannine Coronna** has nothing to disclose.

**Claudine Kiffer** has nothing to disclose.

**Terry Logan, CHCP,** has nothing to disclose.

**Pamela Hallquist Viale, RN, MS, CNS, ANP,** has nothing to disclose.

**Content Reviewers**

**Glenn Bingle, MD, PhD, FACP,** has nothing to disclose.

**Margaret M. Fields, MSN, ACNP-BC, AOCNP®,** has nothing to disclose.

**Allison A. Muller, PharmD, D.ABAT,** has nothing to disclose.

**Wendy J. Smith, ACNP, AOCN®,** has nothing to disclose.

## Disclaimer

This activity has been designed to provide continuing education that is focused on specific objectives. In selecting educational activities, clinicians should pay special attention to the relevance of those objectives and the application to their particular needs. The intent of all Meniscus Educational Institute educational opportunities is to provide learning that will improve patient care. Clinicians are encouraged to reflect on this activity and its applicability to their own patient population.

The opinions expressed in this activity are those of the faculty and reviewers and do not represent an endorsement by Meniscus Educational Institute of any specific therapeutics or approaches to diagnosis or patient management.

## Product Disclosure

This educational activity may contain discussion of published as well as investigational uses of agents that are not approved by the US Food and Drug Administration. For additional information about approved uses, including approved indications, contraindications, and warnings, please refer to the prescribing information for each product.

## How to Earn Credit

To access the learning assessment and evaluation form online, visit www.meniscusce.com

**Statement of Credit:** Participants who successfully complete this activity (including scoring of a minimum of 70% on the learning assessment and complete and submit the evaluation form with an E-mail address) will be able to download a statement of credit.

## ABSTRACT

Approximately 5% to 10% of breast cancer cases and 11% to 18% of ovarian cancer cases are a result of a mutation in the *BRCA1* and *BRCA2* genes, known as hereditary breast and ovarian cancer (HBOC). An inherited mutation in either of these genes increases the probability of malignant transformation and cancer. This article provides a clinical overview of HBOC as well as risk-reduction measures that have the potential to decrease cancer development. The review of the literature highlights the psychological consequences, prophylactic measures, and potential postoperative complications. An examination of this public health issue increases our understanding of the challenges and decision-making processes faced by women with HBOC. Risk-reducing measures and effective strategies that can be implemented to assist these women and their families are discussed. Practice and research implications are outlined to improve health outcomes for these women. Patients’ rights as well as the costs associated with HBOC are also addressed.

## ARTICLE

Approximately 5% to 10% of breast cancer cases and 11% to 18% of ovarian cancer cases are a result of a mutation in the *BRCA1* and *BRCA2* genes, otherwise known as hereditary breast and ovarian cancer (HBOC; American Cancer Society [ACS], 2014; Campeau, Foulkes, & Tischkowitz, 2008; National Cancer Institute [NCI], n.d; Pal et al., 2005, Venkitaraman, 2002; Walsh et al., 2011). Normally, the proteins produced by the *BRCA1* and *BRCA2* genes prevent cells from becoming malignant by aiding in the repair of mutations in other genes through a process known as double-stranded DNA repair. Therefore, an inherited mutation in either of these genes, also known as tumor-suppressor genes, greatly increases the probability of malignant transformation and cancer (NCI, n.d.).

The purpose of this article is to provide a clinical overview of HBOC as well as risk-reduction measures that have the potential to decrease the probability of the development of these cancers. The review of the literature highlights the psychological consequences that can be associated with having a *BRCA* mutation. Prophylactic measures and potential postoperative complications are discussed. An examination of this public health issue allows advanced practitioners (APs) to understand the challenges and decision-making processes faced by women with HBOC. Risk-reducing measures and effective strategies that can be implemented to assist these women and their families are identified. Practice and research implications are outlined to improve health outcomes for these women. Patient rights as well the costs associated with HBOC are also summarized.

## HEREDITARY BREAST AND OVARIAN CANCER

Hereditary breast and ovarian cancer is an inherited genetic condition associated with a mutation in the *BRCA1* or *BRCA2* gene. These genes produce tumor-suppressor proteins that help repair damaged DNA and aid in the stability of a cell’s genetic material. Mutations in the *BRCA1* or *BRCA2* gene are transmitted in an autosomal-dominant pattern in a family, meaning a mutation needs to occur in only one copy of the gene to increase a person’s risk of developing cancer. Therefore, each child of a parent who carries a mutation in one of these genes has a 50% chance of inheriting the mutation, consequently increasing the risk for cancer. Equally, the child also has a 50% chance of not inheriting the mutation; in that case, the risk for cancer becomes comparable to that of the general population.

A woman’s risk for breast cancer increases to 45% to 65% by age 70 if she carries a mutation in either the *BRCA1* or *BRCA2* gene (Centers for Disease Control and Prevention [CDC], 2014; Chen & Parmigiani, 2007; NCI, 2015). Mutations in the *BRCA1* gene increase ovarian cancer risk to 39% by age 70, and *BRCA2* gene mutations increase ovarian cancer risk to 10% to 17% by age 70 (CDC, 2014; Chen & Parmigiani, 2007; NCI, 2015).

There are other cancers associated with HBOC (Petrucelli, Daly, & Feldman, 2010). However, for purposes of this article, only breast and ovarian cancers and their association with HBOC will be discussed. Table 1 lists the types of other cancers associated with each *BRCA* gene.

**Table 1:**
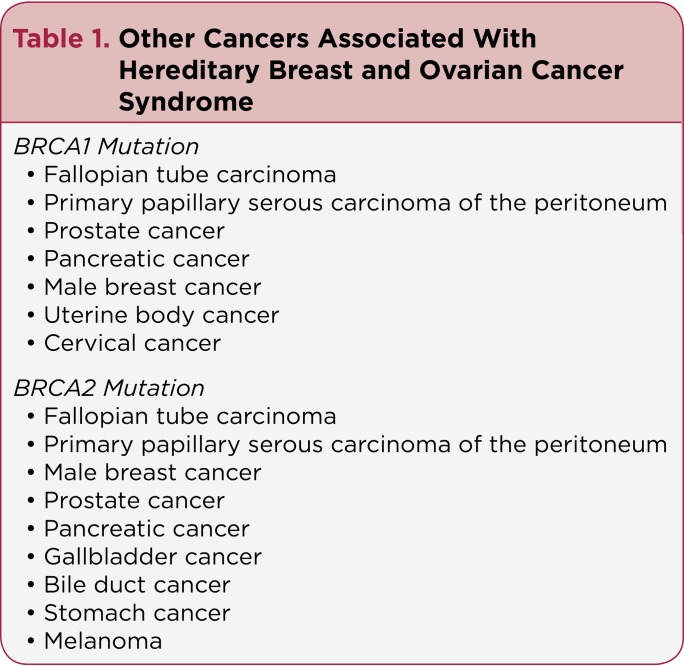
Other Cancers Associated With Hereditary Breast and Ovarian Cancer Syndrome

Genetic tests are available to screen for *BRCA1* and *BRCA2* mutations. The test requires a blood or buccal sample. Genetic counseling should be performed before pursuing screening and after the results are available (National Cancer Comprehensive Network [NCCN], 2015). An increased likelihood of HBOC is suspected based on certain personal and family history characteristics and various clinical criteria. The NCCN has outlined criteria for further genetic risk evaluation (Table 2) as well as criteria for genetic testing (Table 3).

**Table 2:**
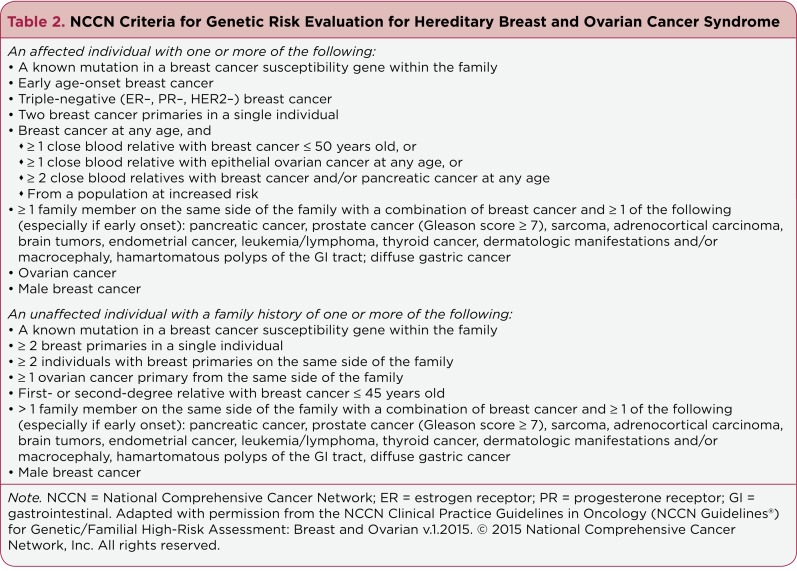
NCCN Criteria for Genetic Risk Evaluation for Hereditary Breast and Ovarian Cancer Syndrome

**Table 3:**
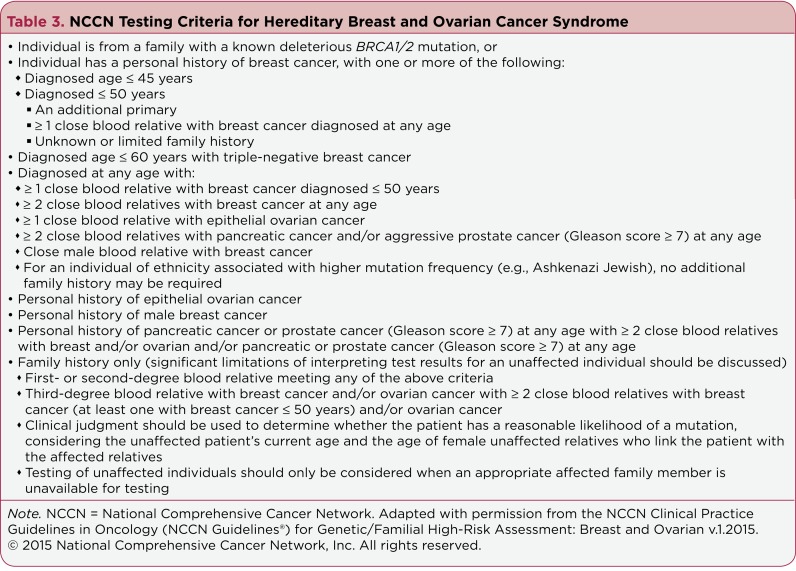
NCCN Testing Criteria for Hereditary Breast and Ovarian Cancer Syndrome

**Risk-Reduction Measures for HBOC**

Risk reduction for breast and ovarian cancers in those with an identified *BRCA* mutation includes both nonsurgical and surgical prophylactic options. Surveillance of breast cancer involves monthly breast self-examinations, clinical breast examinations once or twice a year starting at age 25, and yearly mammograms and magnetic resonance imaging (MRI) of the breast beginning at age 25 (Balmana, Diez, & Castiglione, 2009; NCCN, 2014). Surveillance methods for ovarian cancer may include transvaginal ultrasound, blood tests for CA-125 antigen, and clinical exams every 6 months starting at age 30 or 5 to 10 years before the earliest age of first diagnosis in the family (NCCN, 2014).

*Chemoprevention:* Tamoxifen and raloxifene have been used as "chemoprevention medications" for the reduction of breast cancer in those with a *BRCA* mutation (Balmana et al., 2009; Pruthi, Gostout, & Lindor, 2010). In a study by King et al. (2001), 19 of the 288 cases inherited a deleterious *BRCA* mutation. The study reported a 62% reduction in breast cancer with prophylactic tamoxifen in healthy *BRCA2*-mutation carriers, but there was no reduction in breast cancer incidence among women with an inherited *BRCA1* mutation. These results may be related to the greater chance of development of estrogen receptor–negative tumors in *BRCA1*-mutation carriers relative to *BRCA2*-mutation carriers (NCCN, 2014). The study was limited due to its small size of 19 participants, accounting for only 7% of the study population. Another limitation was that the study did not match its cases and controls based on oophorectomy status.

A second study performed by Gronwald et al. (2006) observed a 50% reduction in contralateral breast cancer in carriers of both *BRCA1* and *BRCA2* mutations when tamoxifen was given as treatment for the initial breast cancer diagnosis. Due to the limitations of the study design, which was case controlled, and the small size of the subgroups, the optimal duration of tamoxifen for chemoprevention could not be determined.

Advanced practitioners should be aware that tamoxifen use is currently approved for risk reduction in both premenopausal and postmenopausal women; however, raloxifene is approved for risk reduction in postmenopausal women only (NCCN, 2014). Additionally, practitioners should warn individuals of the major risks of both raloxifene and tamoxifen, including uterine cancer, blood clots, and stroke (ACS, 2013a).

*Oral Contraceptives:* The use of oral contraceptives has demonstrated a reduction in the risk of ovarian cancer by up to 45% to 50% in *BRCA1*-mutation carriers and up to 60% in *BRCA2*-mutation carriers (McLaughlin et al., 2007). Whittemore et al. (2004) revealed that the longer duration of oral contraceptive use seemed to correlate with a reduction in ovarian cancer.

Studies of the risk of breast cancer with the use of oral contraceptives have yielded inconclusive results. Narod et al. (2002) revealed that among *BRCA1*-mutation carriers, the risk of developing breast cancer was increased; however, this risk was not seen in *BRCA2* carriers. In a case-controlled study by Lee et al. (2008), the use of oral contraceptives in *BRCA*-mutation carriers was not associated with an increased risk of breast cancer.

*Hormone Replacement Therapy:* The effect of hormone replacement therapy (HRT) on breast cancer risk has been examined in clinical studies. In the prospective study by Rebbeck et al. (2005), mutation carriers who underwent risk-reducing salpingo-oophorectomy (RRSO) had a significant decrease in breast cancer risk. When carriers for the *BRCA* mutation without RRSO or HRT were used as the referent group, HRT after RRSO was found not to significantly change the breast cancer risk associated with RRSO.

A case-controlled study by Eisen et al. (2008) studied postmenopausal women who carried a *BRCA1* mutation to compare the risks of breast cancer among those who used HRT and those who did not. A decrease in breast cancer risk was observed among those who took HRT compared with those who did not.

*Risk-Reducing Mastectomy:* The NCCN (2014) recommends discussion of risk-reducing bilateral mastectomy (RRM) for those with a *BRCA* mutation. RRM has shown to substantially reduce the risk of breast cancer (NCI, n.d., NCCN, 2014). Two options for mastectomy are available: total (simple) mastectomy and subcutaneous mastectomy. A total mastectomy removes the entire breast, whereas a subcutaneous mastectomy preserves the nipple-areolar complex.

In a prospective study of 2,483 women with *BRCA1* or *BRCA2* mutations, no breast cancers were diagnosed in the women who underwent RRM during 3 years of prospective follow-up (Domchek et al., 2010). In contrast, 7% of women without RRM over the same follow-up period were diagnosed with breast cancer.

Similarly, in the prospective trial conducted by Heemskerk-Gerritsen et al. (2013), a sample of 570 *BRCA*-positive women underwent RRM or surveillance. Of those women, 57 breast cancer cases occurred in the surveillance group compared with 0 cases in the surgery group.

Rebbeck et al. (2004) also supported the benefit of RRM in *BRCA*-positive patients in their study. In this study, the researchers observed breast cancer in 2 of 191 women after RRM, compared with 184 of 378 women who retained their breasts. This conferred an approximate 95% reduction in breast cancer risk in *BRCA1*- and *BRCA2*-mutation carriers. Furthermore, Skytte et al. (2011) demonstrated an 82% reduction in the risk of breast cancer in their cohort of women who underwent RRM.

For newly diagnosed breast cancer patients who are at risk for carrying a deleterious *BRCA* mutation, knowledge of these results can influence the management of local breast cancer treatment decisions. Genetic testing for high-risk individuals at the time of their breast cancer diagnosis is considered an option for these individuals (Schwartz et al., 2005). Women who undergo testing at the time of their diagnosis and are found to carry a mutation may consider RRM vs. unilateral mastectomy or breast-conserving therapy to decrease their cancer risk (Nusbaum, Peshkin, DeMarco, & Goodenberger, 2009).

In a sample of 35 women considered to be at high risk for carrying a mutation, 32 decided to proceed with genetic analysis. Of these women, seven were positive for a mutation. All seven women opted to have RRM to decrease their cancer risk (Weitzel et al., 2003).

A larger study by Schwartz et al. (2004) revealed that of 194 participants, 167 chose to be genetically tested at the time of their breast cancer diagnosis. Of the 167 participants, 31 were determined to be positive, and 15 of these individuals elected to have RRM as their definitive breast cancer surgery.

*Risk-Reducing Salpingo-oophorectomy:* Risk-reducing salpingo-oophorectomy involves removal of the ovaries and fallopian tubes (ASCO, 2013). The NCCN (2014) recommends RRSO ideally between 35 and 40 years of age, and upon completion of child bearing, or individualized based on the earliest age of onset of ovarian cancer in the family. Risk-reducing salpingo-oophorectomy is associated with an ovarian risk reduction of approximately 70% to 90% and reduction in breast cancer of at least 50% or greater (Maxwell & Domchek, 2012).

A prospective follow-up study compared *BRCA*-positive women who did not undergo RRSO vs. those who did (Domchek et al., 2010). The incidence of ovarian cancer in the nonsurgery group was 6% vs. 1% in the surgery group, demonstrating an approximate 85% reduction in the risk of ovarian cancer with prophylactic surgery.

Confirming the benefit of RRSO, Rebbeck et al. (2002) revealed a 96% risk reduction in ovarian cancer and a 53% risk reduction in breast cancer in 551 women with a *BRCA1* or *BRCA2* mutation. In a follow-up study performed by Finch et al. (2006), RRSO was associated with an 80% overall reduction in ovarian and fallopian tube cancers in women with a known *BRCA* mutation.

The smaller risk reduction for breast cancer with RRSO compared with RRM is associated with ovarian production of estrogen in stimulating hormone-positive breast cancers. Only 10% to 24% of *BRCA1*-associated breast cancers are estrogen receptor–positive, whereas 65% to 79% of *BRCA2*-associated breast cancers are positive for this receptor (Lakhani et al., 2002). Since the ovaries are responsible for the production of estrogen, RRSO would confer a reduction in the risk of estrogen-driven breast cancer. In the population of *BRCA*-positive breast cancers that are estrogen negative, RRSO would not confer a decreased breast cancer risk in this subset of individuals (Kauff et al., 2008).

## PSYCHOLOGICAL CONSEQUENCES

Although learning of one’s *BRCA* testing results may motivate an individual to implement interventions toward prevention of breast and ovarian cancers, discovery of these results can lead to adverse psychological outcomes. Individuals pursuing genetic testing often believe they are at high risk for developing breast or ovarian cancer as well as passing on the mutation to their family members. Testing is performed to alleviate feelings of uncertainty, assist in decision-making regarding prophylactic treatment, and aid family members in preventive care (Andrews, Meiser, Apicella, & Tucker, 2004; Lobel, Dias, & Meyer, 2005). Discovering one has a mutation in the *BRCA1* or *BRCA2* gene can elicit a variety of different emotions, both positive and negative.

Anxiety, distress, and worry are common psychological consequences experienced by women after discovery of their *BRCA*-positive mutation status. A study conducted by Francke et al. (2013) assessed the levels of anxiety in a sample of 32 individuals after learning they were *BRCA*-positive. Of this sample, none was extremely anxious (e.g., cried, lost sleep), 13% were moderately anxious (e.g., couldn’t stop thinking about the result), and 28% were somewhat anxious (e.g., initial disappointment, transient).

Supporting these findings, a study performed by Koehly et al. (2008) revealed increased levels of worry. This worry was associated with the risk of the development of breast cancer and not of ovarian cancer. Patients with an established diagnosis of breast cancer found to have a *BRCA1* or *BRCA2* mutation also may experience feelings of worry. This worry is often related to the chance of cancer recurrence (Wevers et al., 2012).

In the longitudinal study conducted by Reichelt, Heimdal, Moller, and Dahl (2004), distress was exhibited in a sample of 214 women with a known *BRCA1* mutation. Distress was measured prior to disclosure of the individual’s mutation status, 6 weeks after getting the test result, and then 18 months later. Interestingly, there was no significant difference in the levels of distress between each.

However, other researchers have found that mutation carriers sometimes demonstrate changes in their levels of distress over time. Smith et al. (2008) and Beran et al. (2008) revealed greater distress in mutation carriers 1 to 3 months after notification of their results, but this distress resolved after 6 to 12 months. Similarly, a 4-year follow-up of 167 *BRCA*-positive women revealed that only 26% of those individuals were still experiencing distress as a result of their test results (Halbert et al., 2011).

Contrary to the negative feelings associated with having a *BRCA* mutation, positive feelings may also grow from knowing the results. These individuals often experience a sense of empowerment and proactivity in regard to their own and their family’s health and well-being. Upon discovery of their mutation risk, these individuals may be prompted to seek prophylactic mastectomy and/or prophylactic oophorectomy to decrease their cancer risk (Hamilton, 2012; Hamilton, Williams, Skirton, & Bowers, 2009; McCullum, Bottorff, Kelly, Kieffer, & Balneaves, 2007; Hoskins & Werner-Lin, 2011).

For patients with an established diagnosis of breast cancer, knowledge of mutation status unearths concerns of recurrent breast cancer as well as development of another breast cancer. This information may motivate these individuals to seek prophylactic mastectomy to decrease their breast cancer risk (Francke et al., 2013; Hamilton, 2012; Wevers et al., 2012).

Additionally, these individuals identify a sense of concern for family members, especially for their children, in regard to their inherited risk for breast or ovarian cancer. Decisions to pursue prophylactic surgery may be motivated by a woman’s concern of "always being there" for her children. Such individuals may have a heightened awareness of their own mortality, with concerns of leaving their children motherless if they do not pursue measures to decrease their cancer risk (Hamilton, 2012; Hoskins & Werner-Lin, 2011).

## POSTOPERATIVE PHYSIOLOGIC COMPLICATIONS

Although RRM and RRSO can significantly reduce the risk of breast and ovarian cancers for those with a genetic mutation in the *BRCA* gene, there is the potential for both short- and long-term side effects as a result of these surgical procedures (ACS, 2013b; Asante et al., 2010; Parker, 2011). These side effects are outlined in Tables Table 4 and Table 5. Women electing to undergo prophylactic surgery should be educated on the physical side effects of surgery.

**Table 4:**
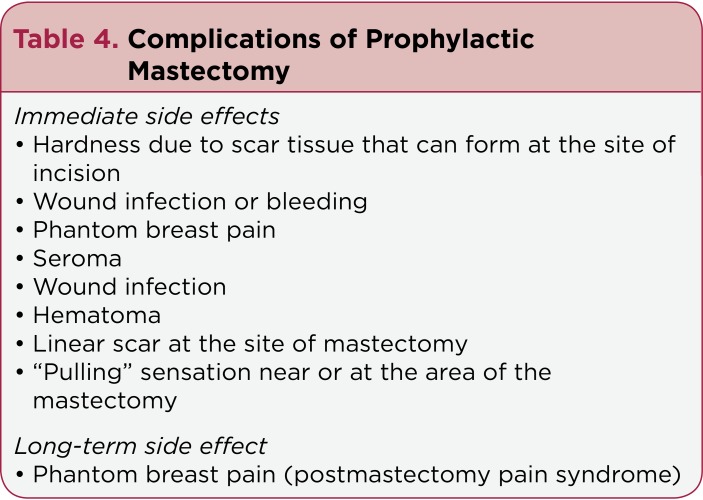
Complications of Prophylactic Mastectomy

**Table 5:**
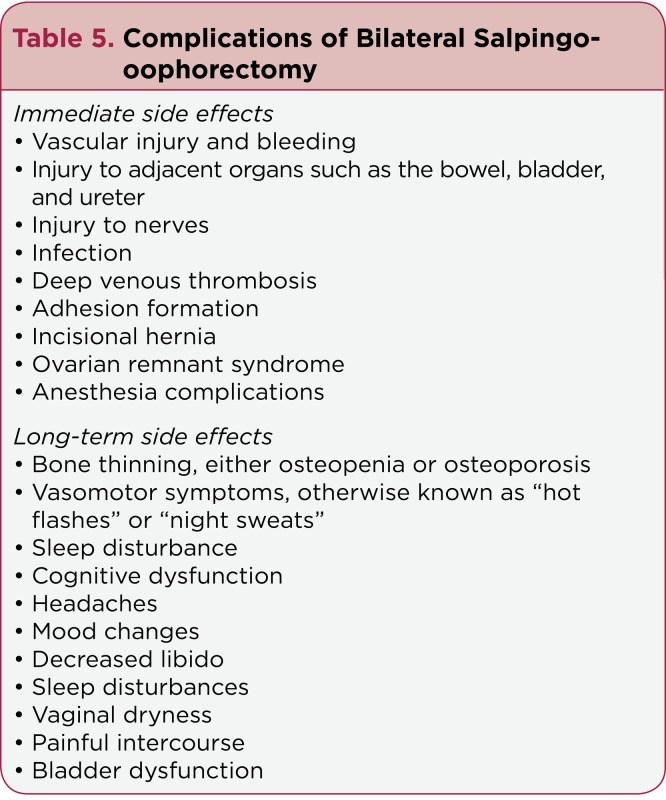
Complications of Bilateral Salpingooophorectomy

Patients undergoing RRM may experience concerns about their body image. Immediate breast reconstruction is a viable option for these individuals. The advantages to immediate breast reconstruction include not waking up to the trauma of losing a breast and eliminating the need for additional surgery. Alternative solutions after mastectomy may include the use of an external prosthesis or a special mastectomy bra (ACS, 2013b).

With RRSO, there is the potential for injury to internal organs such as the bowel, bladder, ureter, blood vessels, and nerves, although these complications are rare. Individuals with prior surgeries, a history of pelvic infection, endometriosis, or other causes of adhesive disease are at greater risk.

## PATIENT CONSIDERATIONS

**Reproductive Options**

Carrying a mutation in the *BRCA* gene can have a significant impact on family planning decisions in those of reproductive age. Reproductive options such as prenatal diagnosis and preimplantation genetic diagnosis (PGD) should be offered to those who are concerned about the mutation status of their future offspring (NCCN, 2014).

Prenatal diagnosis refers to any medical procedure performed to assess the genetic predisposition of a fetus. Methods include amniocentesis and chorionic villous sampling (CVS). These tests pose the risk of miscarriage and potential fetal defects. If the fetus is found to be a carrier of the mutation, couples are confronted with the arduous decision regarding continuation or termination of pregnancy.

Another available option is PGD. This procedure is used to test fertilized embryos for genetic disorders before uterine implantation, thereby offering the option to select unaffected embryos to be transferred to the uterus. This option avoids the risks associated with amniocentesis and CVS and the decision to terminate pregnancy (Offit, Sagi, & Hurley, 2006).

**Family Communication**

Family communication regarding a positive *BRCA*-mutation result can be a complex and arduous process. The complexity can be largely influenced by the psychological outcomes of conveying these findings. Despite its complexity, communication regarding these results is vital so family members are aware of their cancer susceptibility. With this information, family members can pursue the necessary steps to obtain genetic testing. Several studies have examined the communication process between individuals with a positive mutation status and their family members.

Individuals with a *BRCA* mutation are more likely to disseminate this information to their relatives. In the Patenaude and colleagues (2006) cohort sample of 68 women, 92% disclosed this information to their mother and 81%, to their father.

Another study by Finlay et al. (2008) included 115 participants. Of them, 77% disclosed their mutation status to all at-risk family members, whereas 23% disclosed this information to at least one, but not all, at-risk family members. Of these family members, 95% were first-degree relatives, and 78% were second-degree relatives. Similarly, a study by McGivern et al. (2004) found that first-degree relatives were more likely to be informed of a positive mutation status (88%) in comparison to second- and third-degree relatives (45%).

There are many reasons surrounding the motivation to share a positive *BRCA* mutation result with family members. Finlay et al. (2008) and McGivern et al. (2004) revealed several reasons why individuals share their mutation status: a feeling of obligation toward family members, a belief that disclosing risk information will help relatives make medical decisions, a desire to provide relatives risk information, and a desire for relatives to be tested. Another study by Bradbury et al. (2007) cited three major reasons for such disclosure: to provide access to information and awareness, to ensure that their children can be tested, and to explain the family history of cancer.

There are strategies that can be implemented when a *BRCA*-mutation carrier discloses her results to family members. Nevertheless, disclosure of these results should be approached with sensitivity.

Hoskins and Werner-Lin (2011) have recommended several approaches. Conversations should focus on objective information about cancer risk in language understandable to everyone. Personal opinions should only be shared when asked. Hoskins and Werner-Lin (2011) also encouraged providing relatives with printed materials given to them by their health-care professionals. These materials provide a credible source to which family members can refer. They also stress that communication be tailored to individuals’ needs and considerations based on factors such as age, health history, risk tolerance, and coping strategies. After communication is made, family members should be encouraged to meet with a genetics counselor to discuss risks, recommendations for preventive care, and screening.

**Age-Related Issues**

The rate of disclosure of *BRCA* test results to at-risk children is approximated to be 50% (Bradbury et al., 2007; Patenaude et al., 2006; Tercyak et al., 2001). Parental psychological distress often motivates parents to disclose this information to their children; however, disclosure itself does not necessarily alleviate parental distress (Brose et al., 2002). Offspring responses to a parent’s positive *BRCA* mutation can be diverse, and parental support and guidance are salient in these cases. Several studies have examined the patterns of disclosure of mutation results to offspring as well as offspring responses to these results.

For instance, Bradbury et al. (2012) revealed that 41% of offspring of positive parents were disclosed to within 1 month. The median age of offspring was 18 years old. Similarly, a previous study by Bradbury et al. (2007) revealed a 49% rate of disclosure to offspring. Bradbury et al. (2007) noted that most parents disclosed this information immediately; however, 30% reported delayed communication to at least one offspring, ranging from several months to 6 years after receipt of their results. The mean age at disclosure was 18 years old. There were many reasons for the communication delay of results, including waiting for the child to become older, parental adjustment to the information, and taking time to decide how to use the information for themselves and to share in person.

In contrast, Tercyak et al. (2001) found that the mean age of disclosure was 13.5 years old, younger than that observed by Bradbury et al. (2007, 2012). However, the researchers’ mean rate of maternal disclosure was higher at 53%. Barriers to disclosure consisted of factors such as the child being too young or immature and feelings of worry produced in the child.

There are many reasons surrounding parental motivation to disclose mutation results. Disclosure provides the child access to the information and awareness. Subsequently, with this information, children could be tested (Bradbury et al., 2007). Other studies have observed several important factors for disclosure; they include the child’s right to know, a parent’s strong sense of responsibility to disclose, prevention or alleviation of the child’s worry, and promotion of a greater sense of trust and communication between parent and child (Bradbury et al., 2007; Tercyak et al., 2001).

Studies have also looked at the reaction of children to the disclosure of such results. In one study, 30% had an understanding of the significance of the results, whereas 39% did not. Of these participants, 44% had no reaction, no concern, or remained calm, and 26% were scared, angry, or shocked (Bradbury et al., 2007). A later study by Bradbury et al. (2012) revealed responses of concern (28%) or neutrality (25%) followed by feelings of distress and avoidance (18%).

**Patient Education**

Patient education regarding the concept of HBOC syndrome, genetic screening, and testing can be a complex task. Referral to a genetics counselor is deemed necessary for those at risk for HBOC syndrome.

As defined by the National Society of Genetics Counselors (NSGC, 2005), genetic counseling is the process of assisting individuals to understand and adapt to the medical, psychological, and familial implications of genetic contributions to disease. These individuals are specially trained to interpret family and medical histories to assess the risk of disease occurrence or recurrence. Genetic counseling is a vital component of the HBOC risk-assessment process.

According to the NCCN (2014), genetic counselors educate individuals about the genetic, biologic, and environmental factors surrounding the risk of disease or an individual’s cancer diagnosis. This process promotes empowerment of the individual to make educated, informed decisions about genetic testing, cancer screening, and cancer prevention. Presentation of information should be tailored to the age and education of the person undergoing counseling, the individual’s personal exposure to the disease, the level of risk, and the social environment (Trepanier et al., 2004).

Genetic counseling involves both a pretest and posttest session with the individual. Pretest counseling should incorporate a discussion of why HBOC testing is being recommended and how the test results may impact medical management. Furthermore, the cancer risks associated with the specific gene mutation, the possibility of different test results, cost, and the likelihood of a positive result should be discussed. Confidentiality issues should encompass an explanation of the Genetic Information Nondiscrimination Act (GINA; NCCN, 2014).

Posttest counseling is performed to disclose test results. An interpretation of the test results and an assessment of the emotional and behavioral responses of the individual are also performed. The impact of the results on medical management and how the patient will be followed are discussed. Lastly, education about the dissemination of results to family members is emphasized (NCCN, 2014).

## IMPLICATIONS FOR THE ADVANCED PRACTITIONER

**Clinical Practice**

The discovery that one is a carrier of the *BRCA1* or *BRCA2* mutation can elicit a variety of mixed emotions. Individuals in this position are confronted with the concerns that they are at a higher risk for developing breast or ovarian cancer. Adding to these individuals’ stress, there is the worry that this mutation may also affect their family members.

Although understanding the complex psychological processes that this population experiences is essential, APs must be able to look beyond the lens of the patient and identify the needs of family members. Family members who carry a mutation carry as much risk as the affected individual and should be screened as high risk until proven otherwise. By understanding the psychological responses of these women and their families as well as their perception of their cancer risk, APs can provide emotional support and direction as these individuals decide on risk-reducing strategies. Advanced practitioners may need to refer these individuals to a professional counselor or psychologist. Referral to a support group may also be beneficial, as these individuals can discuss their fears and anxieties with others who share the same emotions.

Prior to genetic testing and again upon receipt of an individual’s *BRCA* status, APs should also direct these individuals for genetic counseling, as recommended by NCCN and the US Preventive Services Task Force (USPSTF, 2013). Genetic counselors are responsible for explaining the meaning of the individual’s genetic results with regard to their risks of occurrence of breast or ovarian cancer. The counselors will also guide these individuals on risk-reducing management measures with sensitivity to their family goals as well as their ethical and religious standards. By providing optimal psychological support and education, health-care professionals support these individuals to make informed decisions regarding risk-reducing strategies with which they feel comfortable.

With the growth and advancement of genomic medicine, the need for genetic counseling and testing has also expanded. Advanced practitioners should ideally have some professional training in genetics when caring for patients at high risk for developing cancer due to a genetic mutation. Although APs have the opportunity to become certified in genetics to care for this population of patients, those without genetic certification can still be involved in the care of these patients with appropriate training and education. Advanced practitioners with this background can offer these patients comprehensive optimal care as they go through the genetics process. Table 6 provides information on training and educational resources for health-care providers.

**Table 6:**
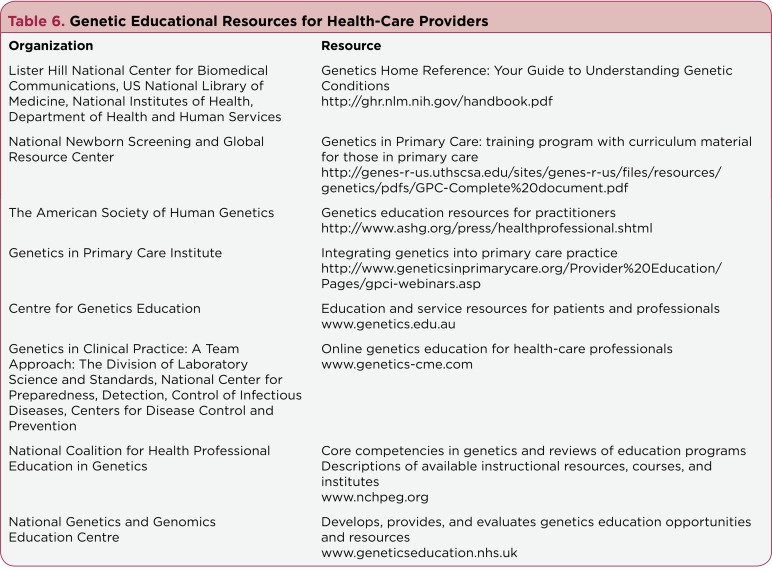
Genetic Educational Resources for Health-Care Providers

**Current Research**

Current research on the psychological consequences of a *BRCA* mutation is concentrated on individuals who are carriers of the *BRCA* mutation; however, there is little research focusing on the needs of family members of the affected individuals. Further research is needed to target this group of individuals so that appropriate interventions can be performed to meet their physiologic and psychological needs.

Because women are now beginning to seek more preventive measures to decrease their cancer risk, in large part based on the influence of the media, a more multidisciplinary effort to guide these individuals may make this journey less complex for them. Current research is focused on the clinician’s role in supporting these individuals. Further research is warranted to examine the benefits of the integration of other disciplines such as social workers, financial counselors, chaplain personnel, genetic counselors, and pharmacists. The integration of different disciplines will ensure that the practical needs of these individuals are also met, including insurance issues, financial coverage, medication education on chemoprevention, and spiritual guidance.

**Patients’ Rights**

Laws and policies exist to govern the rights of *BRCA*-positive women. Because APs are often the first point of contact for them, having an accurate knowledge of this information is essential in ensuring that these women understand their rights.

The Health Insurance Portability and Accountability Act (HIPAA) acknowledges genetic information as protected health information (PHI) and indicates the protection for the confidentiality of PHI. This Act also provides restrictions on health-related information in making coverage decisions and in setting premiums by group health insurers. Furthermore, HIPAA states that genetic information in the absence of a diagnosis cannot be considered a preexisting condition. In the federal government, executive departments and agencies are prohibited from using protected genetic information as a basis for employment decisions (US Department of Health and Human Services, n.d.).

The GINA of 2008 (GINA, 2008) prohibits a group or individual health insurer from discriminating against someone based on results from any type of genetic testing. These results cannot be used as a preexisting condition, nor can they be used to set rates or deny coverage for any services. This Act also prohibits genetic discrimination by employers but does not extend to long-term care insurance, life insurance, or disability insurance.

The USPSTF (2013), composed of an independent group of national experts in prevention and evidence-based medicine, has developed final recommendations on risk assessment, genetic counseling, and genetic testing for *BRCA*-related cancer. The group recommends that primary care providers screen women who have family members with breast, ovarian, fallopian tube, or peritoneal cancer with one of several screening tools designed to identify a family history that may be associated with an increased risk for a mutation in the *BRCA1* or *BRCA2* gene (B recommendation). After receipt of the genetic test results, if a woman is found to be a carrier of a *BRCA* mutation, the USPSTF also recommends that she meet with a genetic counselor to understand her results as well as the implications and treatment options available to decrease her risk of breast and ovarian cancers (USPSTF, 2013).

**Cost of Testing**

Because APs are often the first point of contact for patients, it is important that they are cognizant of the cost involved with genetic testing. A complete *BRCA* analysis is approximated to cost between $1,500 and $4,000 without insurance coverage, with single-mutation site testing ranging from $300 to $400. Most insurance companies will cover the *BRCA* test provided an individual meets the guidelines set by NCCN to test for HBOC. Depending on each insurance company and its method of coverage, the cost may vary from individual to individual. Laboratories offer payment plans as well as financial hardship programs for uninsured and underinsured individuals.

## CONCLUSION

The psychological and physiologic consequences of discovering one carries a *BRCA* mutation can be overwhelming. These individuals are confronted with the decision as to whether or not to pursue prophylactic surgery to decrease their cancer risk. Fear, anxiety, distress, and worry are often felt when patients are struggling with this decision. These emotions are associated with the concerns of developing breast or ovarian cancer as well as the complications and long-term effects of risk-reducing surgery.

Advanced practitioners play an essential role in educating this population about their risk of breast and ovarian cancers as well as measures to decrease this risk. Furthermore, the role of APs is also vital in providing these women emotional support with appropriate referral to a psychologist or support group as necessary. More importantly, APs should also be aware of the policies governing an individual’s rights, specifically with regard to insurance coverage, privacy, and preventive treatment. Being aware of this information will allow APs to accurately direct and educate this population of women so they are fully aware of the rights associated with carrying a *BRCA* mutation.
